# Towards modelling tick-virus interactions using the weakly pathogenic *Sindbis* virus: Evidence that ticks are competent vectors

**DOI:** 10.3389/fcimb.2024.1334351

**Published:** 2024-03-19

**Authors:** Yanan Wang, Zhengmao Xu, Houshuang Zhang, Yongzhi Zhou, Jie Cao, Yuqiang Zhang, Zedong Wang, Jinlin Zhou

**Affiliations:** ^1^ Key Laboratory of Animal Parasitology of Ministry of Agriculture, Shanghai Veterinary Research Institute, Chinese Academy of Agricultural Sciences, Shanghai, China; ^2^ State Key Laboratory of Genetic Engineering, School of Life Sciences, Fudan University, Shanghai, China; ^3^ Center of Infectious Diseases and Pathogen Biology, Key Laboratory of Organ Regeneration and Transplantation of the Ministry of Education, The First Hospital of Jilin University, Jilin, China

**Keywords:** tick, SINV, programmed cell death, innate immunity, tick-virus interactions

## Abstract

Most tick-borne viruses (TBVs) are highly pathogenic and require high biosecurity, which severely limits their study. We found that Sindbis virus (SINV), predominantly transmitted by mosquitoes, can replicate in ticks and be subsequently transmitted, with the potential to serve as a model for studying tick-virus interactions. We found that both larval and nymphal stages of *Rhipicephalus haemaphysaloides* can be infected with SINV-wild-type (WT) when feeding on infected mice. SINV replicated in two species of ticks (*R. haemaphysaloides* and *Hyalomma asiaticum*) after infecting them by microinjection. Injection of ticks with SINV expressing enhanced Green Fluorescent Protein (eGFP) revealed that SINV-eGFP specifically aggregated in the tick midguts for replication. During blood-feeding, SINV-eGFP migrated from the midguts to the salivary glands and was transmitted to a new host. SINV infection caused changes in expression levels of tick genes related to immune responses, substance transport and metabolism, cell growth and death. SINV mainly induced autophagy during the early stage of infection; with increasing time of infection, the level of autophagy decreased, while the level of apoptosis increased. During the early stages of infection, the transcript levels of immune-related genes were significantly upregulated, and then decreased. In addition, SINV induced changes in the transcription levels of some functional genes that play important roles in the interactions between ticks and tick-borne pathogens. These results confirm that the SINV-based transmission model between ticks, viruses, and mammals can be widely used to unravel the interactions between ticks and viruses.

## 1 Introduction

Ticks are obligate blood-feeding arthropods and the most important vectors of pathogens after mosquitoes (Dantas-Torres et al., 2012). Ticks can transmit a wide range of pathogens, including viruses, parasites, and bacteria (de la Fuente et al., 2008; Ferreri et al., 2014; Solano-Gallego et al., 2016; Krause et al., 2019; Rashid et al., 2019; Siddique et al., 2020) that have harmful effects on both human health and animal husbandry (Parola and Raoult, 2001; Grisi et al., 2014; De Meneghi et al., 2016; Ghosh et al., 2019; Ghafar et al., 2020).

Ticks have been reported to transmit more than 35 viruses from multiple viral families (Fang et al., 2015; Mansfield et al., 2017b; Madison-Antenucci et al., 2020). Tick-borne viruses (TBVs) that are pathogenic to humans and animals are primarily from the *Flaviviridae* family and the order of *Bunyavirales*, respectively (Gonzalez, 2014; Kazimirová et al., 2017; Mansfield et al., 2017b). Among them, the louping ill virus of the *Orthoflavivirus* genus is one of the earliest TBVs identified in ticks and is the causative agent of sheep and grouse encephalitis (Wilson, 1946; Bichaud et al., 2014; Shi et al., 2018). Tick-borne encephalitis virus (TBEV) (Mansfield et al., 2009) and Powassan virus (POWV) (Kemenesi and Banyai, 2019) are another two members of *Orthoflavivirus* genus. Both are very harmful zoonotic TBVs (Kaiser, 2008) as they invade the central nervous system of humans and animals, with clinical manifestations including high fever, disorders of consciousness, and even paralysis, resulting in acute nerve damage. Crimean–Congo hemorrhagic fever virus (CCHFV), first reported in the 1960s (Chumakov et al., 1968; Zivcec et al., 2016), belongs to the *Orthonairovirus* genus in the *Nairoviridae* family of the *Bunyavirales*, also known as Xinjiang hemorrhagic fever virus in China, is the most genetically diverse arbovirus among TBVs (Deyde et al., 2006; Bente et al., 2013; Hawman and Feldmann, 2023). It infects humans and most mammals with clinical manifestations including high fever, headache, chills, and bleeding. In severe cases, it can cause multiple organ failure and even death (Whitehouse, 2004; Hawman and Feldmann, 2023).

An increasing number of new viral diseases caused by viruses transmitted by tick bites has been reported (Jongejan and Uilenberg, 2004; Liu et al., 2014; Mansfield et al., 2017b; Wang et al., 2019; Madison-Antenucci et al., 2020; Casel et al., 2021; Dong et al., 2021), including other arboviruses belonging to the genus *Alphavirus* of the *Togaviridae* family. *Sindbis* virus (SINV), a representative of the genus *Alphavirus*, is not a classical TBV but has nevertheless been isolated from ticks of the genus *Hyalomma* near the Mediterranean Sea and in the Arab region (Gresikova et al., 1978; Kostiukov et al., 1981; Scalia et al., 1996; Al-Khalifa et al., 2007). It is commonly found in birds and can be transmitted to other hosts (mammals, birds, reptiles, and amphibians) through mosquitoes. The virus causes myositis and encephalitis in mice and slight fever symptoms in humans (Brummer-Korvenkontio et al., 2002; Adouchief et al., 2016). SINV has a wide range of hosts, replicates rapidly in a variety of host cells, and reaches high titers; therefore, it has been widely used in virology studies (Hernandez and Paredes, 2009; He et al., 2010; Jose et al., 2017; Lasswitz et al., 2022). Recent studies have shown that, similar to severe fever with thrombocytopenia syndrome virus (SFTSV), SINV can replicate in ticks, and both cause similar immune responses in ticks, specifically the activation of the RNAi antiviral response (Xu et al., 2021). The study by Xu et al., 2021 also preliminarily confirmed that SINV has the potential to be used as a model for studying the interactions between ticks and TBVs.

It has been confirmed that the interactions between ticks and tick-borne pathogens (TBPs) are similar to those between other pathogens and arthropods. Pathogens can be recognized in ticks by receptor-ligand binding, as they are in mosquitoes (Beerntsen et al., 2000; Villar et al., 2015; Phelan et al., 2019; Kurokawa et al., 2020). Infection of mosquitoes with dengue virus (DENV), West Nile virus (WNV), and other pathogens destroys the mosquito cytoskeleton (Vlachou et al., 2005; Wang et al., 2010), and cytoskeleton remodeling is also a universal mechanism in the interactions between ticks and TBPs (such as *Borrelia burgdorferi* and *Anaplasma phagocytophilum*) (Cotte et al., 2014; de la Fuente et al., 2016a; Fogaca et al., 2021). Pathogens can interfere with the innate immunity of ticks and facilitate infection (Hajdusek et al., 2013; Fogaca et al., 2021; Maqbool et al., 2022). Knockdown of RNAi-associated proteins Argonaute and Dicer in *I. ricinus*– and *Ixodes scapularis*–derived cell lines resulted in increased LGTV replication and production, proving their role in the tick’s antiviral RNAi response (Ayllon et al., 2015). In tick midguts, *A. phagocytophilum* facilitates and establishes infection through up-regulation of the JAK/STAT pathway (Ayllon et al., 2015). Subolesin and NF-kB protein levels are higher in ISE6 tick cells infected with *A. phagocytophilum* (de la Fuente and Contreras, 2015; Naranjo et al., 2013). Defensin-2 has shown to upregulated both in bacterial and TBEV infection (Chrudimská et al., 2011; Pelc et al., 2014). Apoptosis plays an important role in the infection of *A. phagocytophilum*, as confirmed both *in vivo* and *in vitro* (de la Fuente et al., 2016a). RNA silencing of X-linked inhibitor of apoptosis protein significantly increases tick colonization by the bacterium *A. phagocytophilum* (Severo et al., 2013). Similarly, some TBVs, such as louping ill virus (Johnson, 2017; Oliva Chávez et al., 2017), TBEV (Mansfield et al., 2017a) and SFTSV (Fares and Brennan, 2022) utilize the major cellular pathways (innate immunity, apoptosis and RNAi responses) in mammalian or tick cells to facilitate virus replication. *In vitro*, when an *I. ricinus*–derived cell line was infected with flavivirus, the transcription level of cytochrome C gene was upregulated, which is a molecule known to be associated with apoptosis (Mansfield et al., 2017a). Langat virus (LGTV)-infected ISE6 cells showed an increase in histone protein expression (Grabowski et al., 2016).

Research on TBPs has focused on studying the interactions between ticks and bacteria (Khanal et al., 2017; Taank et al., 2018; Dahmani et al., 2020; Kurokawa et al., 2020; Ramasamy et al., 2020) while we are in the early stages of our understanding of tick-virus interactions (Mansfield et al., 2017a; Damian et al., 2020; Maqbool et al., 2022; Migné et al., 2022). Most TBVs are virulent and the requirements for biosafety research are high, which severely restricts and hinders relevant basic research on TBVs (Kaiser, 2008; He et al., 2010; Maffioli et al., 2014; Adouchief et al., 2016; Grabowski et al., 2016; Grabowski and Hill, 2017; Kazimirová et al., 2017; Mansfield et al., 2017b; Grabowski et al., 2018; Shi et al., 2018; Zhuang et al., 2018; Kemenesi and Banyai, 2019; Salata et al., 2021; Ahmed et al., 2022; Maqbool et al., 2022; Raney et al., 2022).

Currently, there is an urgent need to establish alternative low-pathogenic TBV models that conform to the replication and transmission dynamics of most TBVs in ticks. To address these conditions, utilizing SINV as a model pathogen, we established a series of methods to prove the feasibility of using SINV to study the interaction between ticks and viruses. We believe that the application of SINV-based tick transmission models will provide a new platform for future examination of interactions between ticks and viruses.

## 2 Materials and methods

### 2.1 Ticks and animals

Laboratory colonies of *Rhipicephalus haemaphysaloides* and *Hyalomma asiaticum* were maintained by feeding on New Zealand White rabbits (weighing approximately 3 kg) or 6–8-week-old BALB/c mice, provided by the Shanghai Laboratory Animals Center (Shanghai Institutes for Biological Science, Chinese Academy of Sciences, Shanghai, China) (Zhou et al., 2006). These two species of ticks have been undergoing continuous breeding in our lab for over 10 years. We usually feed ticks on clean grade rabbits to ensure that the ticks are not infected with any other pathogens.

### 2.2 Cells and tissue culture

BHK-21 cells preserved in our laboratory were cultured in Dulbecco’s modified Eagle’s medium (DMEM) supplemented with 8% heat-inactivated fetal bovine serum (Biological Industries, Kibbutz Beit Haemek, Israel) and 1% penicillin-streptomycin. The cells were cultured and passaged in a 5% CO_2_ incubator at 37°C. The tick cell line, ISE8/CTVM-19, from embryos of *Ixodes scapularis*, was maintained in *L-15* (Leibovitz) medium (*L15* medium supplemented with 20% heat-inactivated fetal bovine serum, 10% tryptose phosphate broth, 1% L-glutamine (200 mM), and 1% penicillin-streptomycin at 30°C) (Bell-Sakyi et al., 2007; Weisheit et al., 2015).

Unfed adult *R. haemaphysaloides* were sterilized in 70% ethanol for 30 s and then dried with filter paper. The salivary glands and midguts of each tick were removed by microdissection, immediately transferred to *L15* medium supplemented with 1% penicillin-streptomycin at 30°C, and cultured *in vitro*, as previously described (Grabowski et al., 2017).

### 2.3 Virus culture and infection

BHK-21 cells were cultured in T75 flasks until reaching a density of 70–80%. After washing the cells once with serum-free DMEM, 3 mL of serum-free DMEM was added to each flask, followed by 50 μL of SINV-wild-type (WT) and SINV-eGFP virus (supplied by Dr. Margaret MacDonald from Rockefeller University and Dr. Zhang Yuqiang from Fudan University) solutions at a titer of 1 × 10^7^ (PFU)/mL. The cells were incubated at 37°C for 1 h before the addition of complete DMEM containing 10% fetal bovine serum. Viruses were collected 48 h post-infection. The collected viruses were freeze-thawed three times, concentrated using a column (Amicon Ultra-15 10 kDa centrifugal filter unit; Merk-Millipore, Billerica, MA, USA), and subjected to a viral plaque assay to determine the titer.

Infection with SINV requires a constant neutral pH, which can be achieved by further supplementation with tick basic medium as described above (Bell-Sakyi et al., 2007; Weisheit et al., 2015). A total of 50 μL of SINV-WT virus solution with a titer of 1.2 × 10^9^ PFU/mL was resuspended in 1 mL of tick basic medium, and 500 μL was added to 30–50% confluent tick cell cultures or tick tissues (salivary glands and midguts) (Grabowski et al., 2017, 2019), cultured in a 6-well plate. Infection of CTVM-19 cells with SINV was monitored by quantitative real-time polymerase chain reaction (qRT-PCR) every two days (Bell-Sakyi et al., 2007; Weisheit et al., 2015).

### 2.4 Tick infection by microinjection

The SINV-WT stock solution with a viral titer of 1.2 × 10^9^ PFU/mL was diluted 10- or 100-fold with DMEM. Unfed female ticks were microinjected with 1 μL per *H. asiaticum* and 0.5 μL per *R. haemaphysaloides* to the coxa of the fourth right leg (60 females per group, injected 20 females per dilution, two independent groups). After microinjection, ticks were maintained in an incubator at 24°C and 95% humidity. Ticks at different time points after infection were collected (1 adult tick per sample), and used for determination of the infectious viral particles by plaque assays.

For tissue tropism assays, unfed female *R. haemaphysaloides* (at least 60 ticks) were microinjected with SINV-WT/SINV-eGFP stock solution (0.5 μL per tick) as mentioned above. Different tick tissues (midguts, salivary glands, and ovaries) infected with different virus doses were collected by microdissection at different time points post-infection, and used for the subsequent detections.

### 2.5 Transmission of SINV by *R. haemaphysaloides*

Fifty 3-4-week-old C57 mice were divided into two groups. The mice were intraperitoneally injected with SINV-WT/SINV-eGFP (2 × 10^7^ PFU/mouse) or an equal volume of DMEM as control. Twenty-four hours after injection, mice were infested with different developmental stages of *R. haemaphysaloides* (a mouse was infested with 200 larvae, 50 nymphs, or two adult females and one male ticks). Post-engorgement, the ticks were collected and stored at 24°C in a constant temperature biochemical incubator with 95% humidity for molting. Starting on day 7 after molting, molted nymphs (30 nymphs per sample) and female *R. haemaphysaloides* (one adult tick per sample) were collected at 14 d intervals. qRT-PCR and plaque assays were used to determine whether the collected nymphs or adult ticks were infected with SINV, and the infection rates in adult ticks were calculated. The same batch of ticks that tested positive was fed on healthy 3–4-week-old C57BL/6J mice (each stage of the tick bit at least 3 mice). On the 3^rd^ feeding day, the mice were sacrificed, and different tissues (brain, blood, heart, liver, spleen, and lungs) were collected for SINV detections, as described above.

### 2.6 Virus plaque assays

Samples from the transmission experiments as described above were collected, including the samples of ticks (larvae, nymphs or adult ticks) and different tissue (from ticks or mice) lysates infected with SINV (SINV-WT/SINV-eGFP). Then, 200–500 μL of phosphate-buffered saline (PBS) was added to each sample (for nymphs, 30 unfed nymphs each sample; for adult ticks, one whole tick per sample), and after grinding by tissue crusher, a part of the tick lysate was used for RNA extraction, and the remaining part was used for virus plaque assays. BHK-21 cells were cultured in 6-well plates at a density of 90%. The cells were then washed once with serum-free DMEM. The tick samples were diluted in a 10-fold series (10^-1^–10^-10^) and the appropriate dilution was selected for each sample type (the ticks and tick tissues infected by microinjection are usually diluted in several series because of the high viral dose, whereas samples of ticks from transmission experiments were used as stock solutions directly). The sample suspension (0.5 mL) was inoculated at each dilution, with three replicate wells per dilution. Blank controls of DMEM without the virus were used in three wells. Samples were incubated at 37°C for 1 h and shaken every 20 min. The viral suspension was discarded, and 2 mL of cooled solid medium containing 0.5% low-melting-point agarose (prepared by mixing 4% FBS-containing 2× DMEM culture medium and an equal volume of 1% low-melting-point agarose) was added. Cells were further incubated at 37°C with 5% CO_2_, and changes in cells were continuously observed. When significant cytopathic effects were observed (about 48–72 h after infection), the cells were covered with a second layer of low-melting-point agarose medium containing 10% crystal violet staining solution and incubated at room temperature for approximately 6 h. Next, 1 mL of 4% formaldehyde was added to fix the cells, the supernatant was discarded, the morphology of the plaques was observed, and their number was recorded. The virus titer of the test sample was displayed as PFUs per milliliter: Sample titer (PFU·mL^-1^) = (number of plaques per well × sample dilution)/volume of sample added to each well (mL). The mean viral titer in the supernatant at each time point was calculated.

### 2.7 Transmission electron microscopy analysis

Glutaraldehyde-fixed midguts of female *R. haemaphysaloides* infected with SINV by microinjection were prepared as ultra-thin sections and then subjected to standard TEM procedures (Umemiya-Shirafuji et al., 2008). The sections were then viewed and photographed using a Tecnai G2 Spirit BIOTWIN TEM (FEI, Hillsboro, OR, USA).

### 2.8 RNA extraction and transcriptome of *R. haemaphysaloides* midguts and salivary glands

Adult *R. haemaphysaloides* were divided into two groups (300 female ticks per group, two independent groups). The experimental group ticks were microinjected with SINV-WT (6 × 10^4^ PFU/per tick), while the ticks of control groups were injected with an equal volume of DMEM (0.5 μL/per tick). The midguts and salivary glands of different experimental groups were collected at different time points after microinjection (unfed female ticks infected for 3 days or 9 days) or different time points of blood-feeding (infected female ticks fed for 3 days or 5 days). Midguts and salivary glands of both unfed and fed *R. haemaphysaloides* females were homogenized in TRIzol reagent (Invitrogen, Carlsbad, CA, USA), and purified RNA was used for the construction of paired-end cDNA libraries using a NEBNext^®^ Ultra™ RNA Library Prep Kit (New England Biolabs, Ipswich, MA, USA), according to the manufacturer’s instructions. Sequences were tagged with specific barcodes, and paired-end reads were sequenced using an Illumina HiSeq platform (Illumina San Diego, CA, USA) at the Beijing Genomics Institute (BGI, Beijing, China).

RNA-seq data were cleaned and formatted using an Agilent 2100 Bioanalyzer (Agilent Technologies, Santa Clara, CA, USA). High-quality reads were assembled using the Trinity program with default parameters (Grabherr et al., 2011). The assembled transcripts were extended and clustered using TGICL software (Pertea et al., 2003). Assembled transcripts were processed for functional annotation and classification. The *de novo* approach for transcriptome assembly, TransDecoder (http://transdecoder.sourceforge.net), was used to identify putative protein coding sequences from the contigs. Seven different functional databases (NR, NT, Gene Ontology [GO] terms [Harris et al., 2008], Clusters of EuKaryotic Orthologous Groups [KOG] [Tatusov et al., 2000], Kyoto Encyclopedia of Genes and Genomes [KEGG] pathways [Kanehisa et al., 2004], and SwissProt and InterPro [Zdobnov and Apweiler, 2001]) were used to annotate all assembled transcripts (Unigenes). Differentially expressed genes (DEGs) were identified using the MA-plot-based method with a random sampling model by comparing the unfed library with the engorged library. Genes with a fold change *>* 3 and a *P*-value *<* 0.001 were considered differentially expressed.

### 2.9 qRT-PCRs

The different developmental stages of *R. haemaphysaloides* and tissues from female *R. haemaphysaloides* (midguts and salivary glands) or mice (brain, blood, heart, liver, spleen, lungs and kidneys) were collected for RNA extraction as described above. RNA was converted into first-strand cDNA using a HiScript III RT SuperMix for quantitative polymerase chain reaction (qPCR) (+gDNA wiper) kit (Vazyme Biotech, Nanjing, China) following the manufacturer’s protocols. The double-stranded cDNAs were used as a template for qRT-PCR along with specific primers (**Supplementary Tables S1**, **S2**, **S3**, and **S4**), which were designed using Primer Premier 5 (Premier Biosoft International, Palo Alto, CA, USA). qRT-PCR was performed using the ChamQ Universal SYBR qPCR Master Mix (Vazyme Biotech) green and gene-specific primers on a QuantStudio 5 System (Applied Biosystems, Foster City, CA, USA). The qRT-PCR cycling parameters were 95°C for 30 s, followed by 40 cycles of 95°C for 5 s and 60°C for 30 s. All samples were tested three times. Relative quantification was performed using the 2-^△△Ct^ method (Livak and Schmittgen, 2001, Ginzinger, 2002). In particular, all data were normalized to the expression of elongation factor-1 (ELF1A) (GenBank accession no. AB836665) (Nijhof et al., 2009), and their level of expression expressed relative to that of tissue (midguts or salivary glands from ticks) and cell samples (from CTVM-19 cells).

In the SINV transmission experiment, the absolute qPCR method was employed to determine the viral load in different developmental stages of *R. haemaphysaloides* or the tissues of mice, using primers targeting the non-structural protein 1 (Dahl et al., 2022).

### 2.10 TUNEL staining

Paraformaldehyde-fixed midguts of unfed female *R. haemaphysaloides* infected by microinjection at different time points were subjected to paraffin sectioning and antigen retrieval. The sections were then permeabilized with 0.1% Triton X-100 and incubated for 1 h with 1:9 terminal deoxynucleotidyl transferase mixed with fluorescently-labeled deoxyuridine triphosphate at 37°C, following the instructions of the Roche *In Situ* Cell Death Detection Kit POD (Roche, Mannheim, Germany). To stain the nuclei, sections were washed three times with PBS (0.14 M NaCl, 0.0027 M KCl, 0.01 M phosphate buffer; pH 7.4)/0.5% Tween-20, and then incubated with 1 μg/mL 4′, 6′-diamidino-2-phenylindole (DAPI, Invitrogen) in dd H_2_O for 20 min. After washing, the sections were mounted using a fluorescent mounting medium under glass coverslips, and then viewed and photographed using a Zeiss LSM880 Laser Scanning Confocal Microscope (Carl Zeiss, Jena, Germany).

### 2.11 Western blot

Total proteins from midguts of unfed female *R. haemaphysaloides* infected by microinjection at different time points were extracted using Tris-buffered saline (TBS) (10 mM Tris-HCl, pH 7.5; 150 mM NaCl with 1 mM phenylmethanesulfonyl fluoride). The total concentration of the extracted protein was determined using the Bradford Protein Assay Kit (Beyotime, Shanghai, China) following the manufacturer’s instructions. For SDS-PAGE (12%; Genescript, Nanjing, China), loading of 20 µg protein/well was performed, and proteins on the gel were transferred onto a nitrocellulose membrane. Anti-RhCaspase 7 (Wang et al., 2020), RhATG8 (Wang et al., 2021), and anti-GFP primary antibodies (Cell Signaling Technology, Danvers, MA, USA) were used to detect apoptosis, autophagy, and SINV-eGFP in the protein extracts, and anti-tubulin primary antibodies (Proteintech, Rosemont, IL, USA) were used as constitutive controls to normalize the signal from the target protein. After primary incubation, a goat anti-mouse IgG (H + L) secondary antibody conjugated with HRP (Invitrogen) and IRDye 800CW goat anti-mouse IgG (H + L) (LI-COR, Lincoln, NE, USA) were used as secondary antibodies in the assays. Images were captured using a ChemiDoc Touch (Bio-Rad, Hercules, CA, USA) or Odyssey Imaging System (LI-COR).

### 2.12 Data analysis

GraphPad PRISM 6.0 software (Graph Pad Software Inc., La Jolla, CA, USA) was used for all data analyses. Mean ± standard error (SEM) values were calculated for three independent experiments, and two-tailed Student’s *t* tests were used to identify significant differences between groups (*p < 0.05; **p < 0.01, ***p < 0.001, ****p < 0.0001).

## 3 Results

### 3.1 Transmission of SINV via *R. haemaphysaloides* at different developmental stages

**Figure 1:**
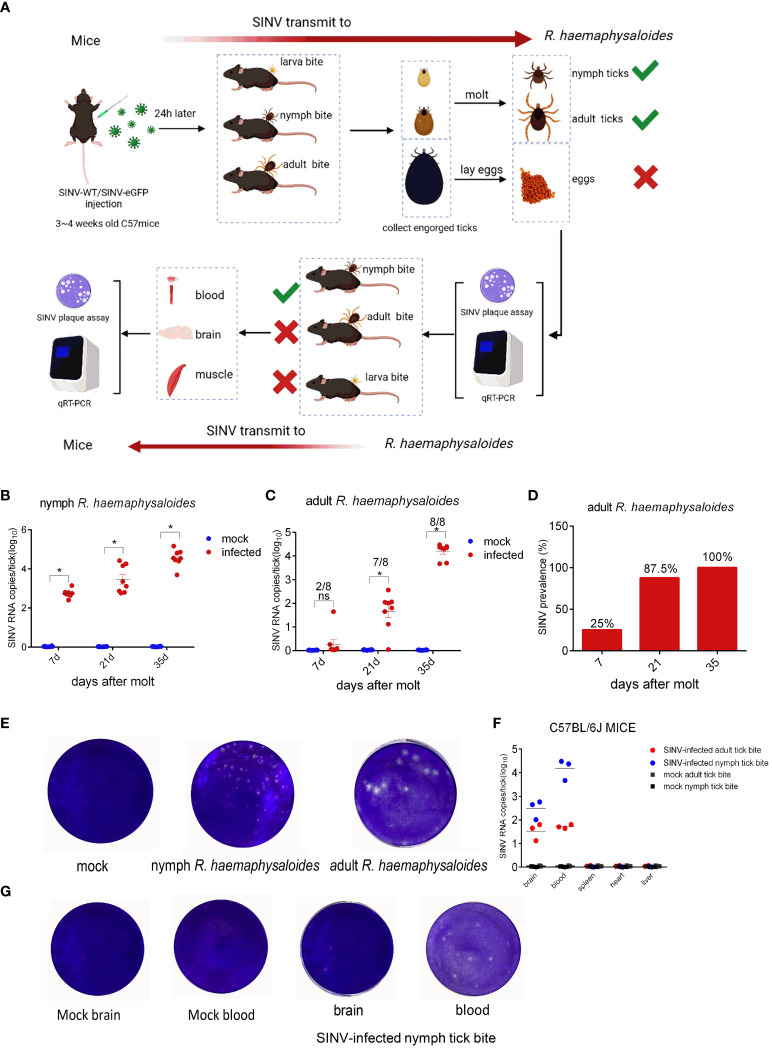
SINV transmission through mouse-tick experiment. **(A)** Schematic diagram of SINV transmission between mice and ticks. **(B)** qRT-PCR detections in *R. haemaphysaloides* nymphs (larval bite) at different time points after molting, with eight replicates per time point and 30 *R. haemaphysaloides* nymphs per sample. **(C)** qRT-PCR detection in adult *R. haemaphysaloides* (nymphal bite) at different time points after molting (1 adult *R. haemaphysaloides* per sample, 4 female and 4 male *R. haemaphysaloides* examined independently at each time point). **(D)** Statistics on the positive rates of molted *R. haemaphysaloides* adults in **(C)**. **(E)** Virus plaque assays of qRT-PCR-positive *R. haemaphysaloides* samples. **(F)** qRT-PCR detections in infected tissues of C57BL/6J mice. For the mice bitten with SINV-infected nymphal *R. haemaphysaloides*, 50 SINV-infected nymphs were attached to the back of three mice. For the mice bitten with SINV-infected adult *R. haemaphysaloides*, one SINV-infected female *R. haemaphysaloides* and one male *R. haemaphysaloides* were attached to the back of mice. Different tissues from mice were collected for SINV-related assays on days 3–4 of tick feeding. **(G)** Virus plaque assay of qRT-PCR-positive mice tissue samples in **(F)**. The viral RNA levels in the transmission experiment were detected and calculated by absolute quantitative PCR. The significance of the differences in **(B)**, **(C)** and **(F)** was determined by the Student's t-test: *p < 0.05, ns means not significant

To demonstrate that SINV could be transmitted via ticks, we attached *R. haemaphysaloides* at different developmental stages (including larval, nymphal, and adult ticks) to SINV- WT/SINV-eGFP-infected mice (**Figure 1A**). Post-engorgement, ticks were collected for molting or oviposition. The results showed that the SINV titer increased over time in both molted nymphal and adult ticks (**Figures 1B, C**). However, no viral RNA was detected in the eggs or hatched larvae laid by engorged female *R. haemaphysaloides* (**Supplementary Figures S1A, B**). As time passed after molting, the virus replicated within the ticks, and the positivity rate in adult ticks (one whole adult tick per sample, molted from engorged nymphs) gradually increased, reaching 100% approximately 5 weeks after molting, as determined by qRT-PCR (**Figure 1D**). We conducted virus plaque assays on qRT-PCR-positive nymphs and adults and found that only samples with high viral RNA copy numbers formed plaques after infecting BHK 21 cells (**Figure 1E**). SINV RNA transcripts were detected in both blood and brain of the mice bitten by SINV-infected nymphs and adult *R. haemaphysaloides* (**Figures 1A, F**). However, the tissue lysates from mice exposed to SINV-infected adult *R. haemaphysaloides* failed to form viral plaques after infecting the BHK-21 cells. Viral plaque formation only occurred in the BHK-21 cells infected with blood and brain lysate samples from mice bitten by SINV-infected nymphal *R. haemaphysaloides* (**Figure 1G**).

### 3.2 Replication curves of SINV with different titers in different ticks

**Figure 2:**
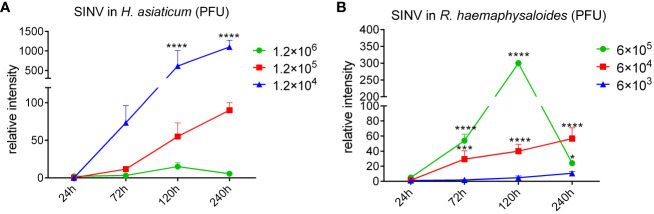
The replication curves of SINV in different tick species. **(A)** The replication curves of SINV in *H*. *asiaticum* by virus plaque assays. **(B)** The replication curves of SINV in adult female *R. haemaphysaloides* by virus plaque assays. 60 females per group, injected 20 females per dilution, two independent groups. For each infection time point, 3 ticks were taken per injection dose, one tick per sample. The injection volume to each female *H. asiaticum* was 1 μL, while the injection volume to each female *R. haemaphysaloides* was 0.5 μL. The PFU of virus at the starting time point was regarded as 1, and the ordinate is the concentration at each subsequent time point which is a multiple of the starting point. Bars represent the mean ± SD of three replicates. Significance of differences as determined by Student’s t-test: *p < 0.05, ***p < 0.001, ****p < 0.0001.

*H. asiaticum* and *R. haemaphysaloides* females were infected with different doses of SINV by microinjection, and we found that SINV could replicate in both species of ticks, and the replication curve with the same virus gradient showed the same trend (**Figure 2**). The preliminary results showed that SINV can be used as a model for studying tick-virus interactions. While viral replication increased with increasing quantity of virus in inoculum until 1.2 × 105 PFU/per tick, it diminished at higher doses (6 × 105 PFU/per tick and 1.2 × 106 PFU/per tick). Based on these results, we selected 6 × 104 PFU/per tick as the infective dose for subsequent experiments (**Figure 2**). Only injected with the appropriate dose of the virus (6 × 10^4^ PFU/per tick and 1.2 × 10^5^ PFU/per tick), the virus continues to replicate within ticks. Based on these results, we confirmed this dosage (6 × 10^4^ PFU/per tick) as the infective dose for the subsequent experiments.

### 3.3 Tissue tropism of SINV in *R. haemaphysaloides*

**Figure 3:**
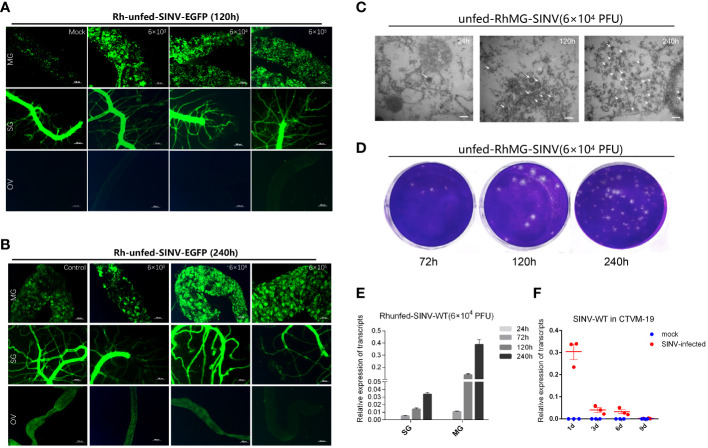
Molecular biology verification of SINV replication in the midguts and salivary glands of unfed female *R. haemaphysaloides in vivo* infected by microinjection. **(A, B)** Distribution of SINV-eGFP in different tissues of female *R. haemaphysaloides* at different infection time points. MG: midgut; SG: salivary gland; OV: ovary; scale bar: 100 μm. **(C)** TEM observation of the number of SINV particles at different infection time points; scale bar: 100 nm. **(D)** Virus plaque detection of SINV at different infection time points in the midguts of unfed female *R. haemaphysaloides.* **(E)** qRT-PCR to verify the replication of SINV in the midguts and salivary glands of unfed female *R. haemaphysaloides*. **(F)** The replication curves of SINV in CTVM-19 cells. Bars represent the mean ± SD of three replicates.

When ticks were infected with different doses of SINV-eGFP, we found that the virus specifically aggregated in the tick midguts during the early stage of infection (120 h after microinjection) (**Figures 3A, B**), whereas aggregation of specific fluorescence was not observed in the other two tissues (salivary glands and ovaries). We also cultured tick tissues *in vitro* and infected them with SINV-eGFP, and confirmed that SINV replicates in both the midguts and salivary glands of ticks (**Supplementary Figures S1C, D**). The specific fluorescence intensity in the midguts of ticks treated with different viral doses increased with increasing infection duration (**Figure 3B**). TEM results showed that the number of viral particles in the tick midguts increased with increasing infection time at the same infection dose (**Figure 3C**). The results of qPCR and virus plaque assays also confirmed that SINV-eGFP mainly accumulated in the tick midguts, and that the amount of virus in the midguts increased significantly with the duration of infection (**Figures 3D, E**). We did not observe specific fluorescent aggregates in tick ovaries after microinjection of SINV (**Figure 3A, B**), and SINV-eGFP failed to replicate in the tick embryonic cell line CTVM-19 (**Figure 3F**).

### 3.4 Global transcriptome profiles of the midguts of unfed female ticks infected with SINV

**Figure 4:**
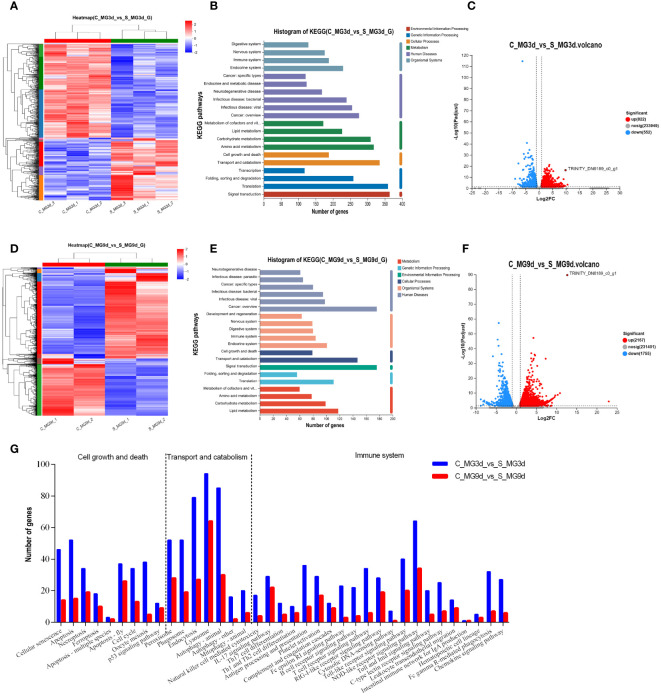
Transcriptome sequencing and DEG analysis of the tick midguts at different time points of SINV infection by microinjection. **(A, D)** Cluster analysis of transcriptome sequencing in the midguts of unfed female *R. haemaphysaloides* at different infection time points after microinjection. Histogram of the KEGG functional annotation of transcriptome DEGs in SINV-infected tick midguts at 3 d **(B)** and 9 d **(E)**. **(C, F)** Volcano plots of DEGs in the midguts of unfed female *R. haemaphysaloides* at different infection time points after microinjection, with upregulated genes shown in red and downregulated genes shown in green. **(G)** Histograms of immune, transport and catabolism, cell growth and death-related genes among the DEGs in the 3 d and 9 d transcriptomes in the midguts of SINV-infected ticks. TRINITY_DN6189_c0_g1 refers to SINV- specific transcripts.

To screen for regulatory molecules associated with SINV replication in tick midguts, we collected unfed tick midguts (infected with SINV-WT by injection for 3 days or 9 days) for transcriptome sequencing and differential analysis with control groups at the same time points. Heat-map clustering analysis of all DEGs revealed that the expression-clustering patterns were reproducible within the different experimental groups and clearly separated from those of the corresponding control samples and SINV-WT-infected samples (**Figures 4A, D**). KEGG annotation analysis of the DEGs at different infection time points showed that the differentially expressed gene composition was broadly similar, but the number of KEGG-annotated DEGs at 3 days of infection was higher than that at 9 days of infection (**Figures 4B, E**). Many of the genes were differentially expressed, with 1374 (a total of 822 upregulated and 552 downregulated DEGs detected) and 3922 (a total of 2167 upregulated and 1755 downregulated DEGs detected) genes differentially expressed at 3 days and 9 days of infection, respectively (**Figures 4C, F**). These results are in contrast to the difference in KEGG-annotated DEGs, demonstrated in **Figures 4B, E**. We detected SINV-specific DEGs (TRINITY_DN6189_c0_g1) in DEG libraries at different time points during infection, and the number of SINV transcripts was significantly greater at 9 days than at 3 days after infection (**Figures 4C, F**). To determine a relevant time point for analysis of tick-virus interactions during SINV viral cycle, we counted the DEGs with widely-recognized pathways (cell growth and death, substance transport and catabolism, and immune system) associated with host resistance to pathogenic infection and found a greater number of associated genes at 3 days of infection (**Figure 4G**).

### 3.5 SINV infection-induced programmed cell death in the tick midgut

**Figure 5:**
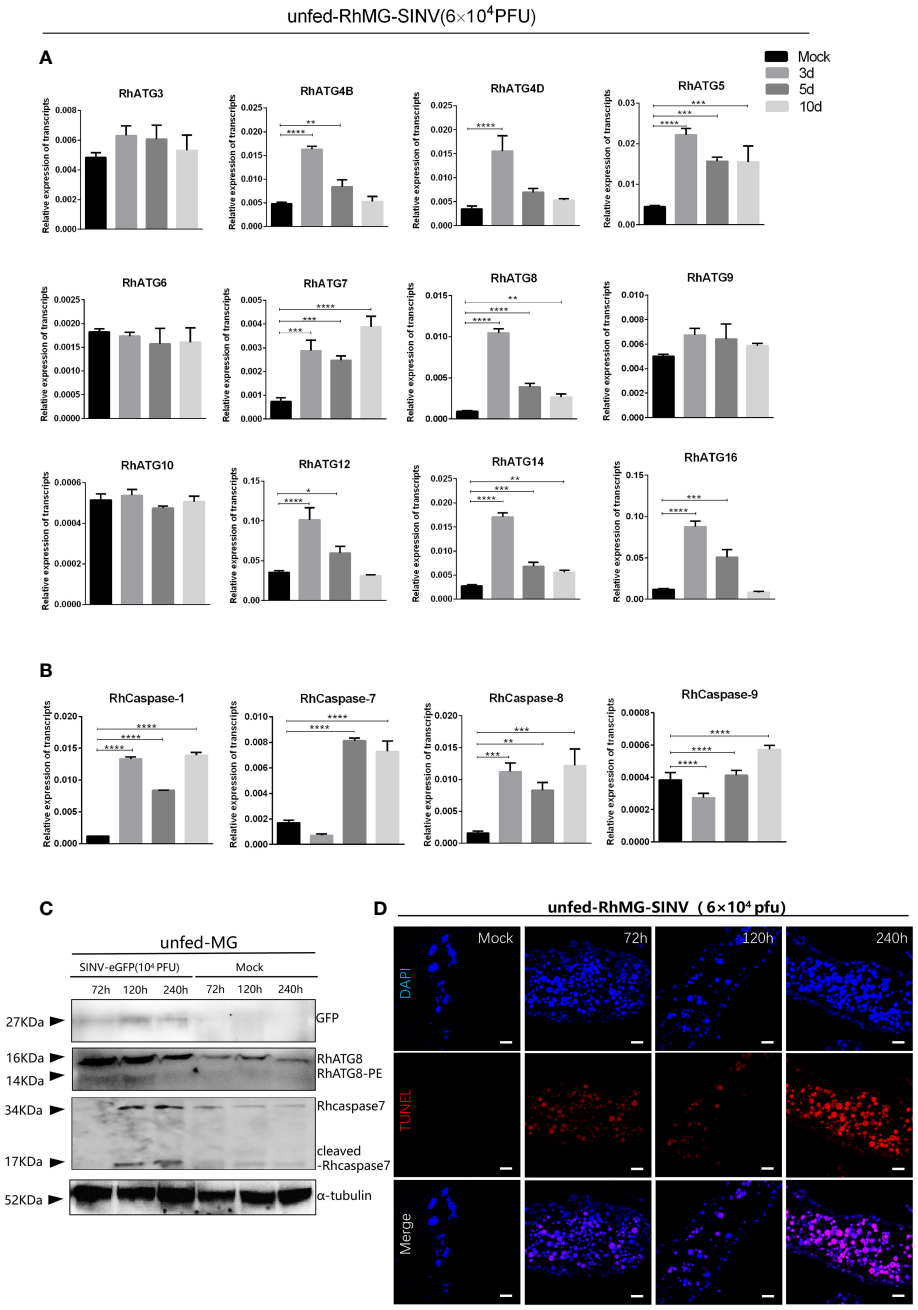
Changes of autophagy and apoptosis levels during SINV replication in the midguts of unfed female *R. haemaphysaloides*. Quantitative qPCR analysis of the expression levels of **(A)** autophagy-related genes and **(B)** apoptosis-related genes after virus infection, bars represent the mean ± SD of three replicates. Significance of differences as determined by Student’s t-test: *p < 0.05, **p < 0.01, ***p < 0.001, ****p < 0.0001. **(C)**Western blot detection of autophagy- and apoptosis-related genes at different viral infection time points. The midguts of unfed female *R. haemaphysaloides* infected at 3, 5, and 10 d were collected, and anti-RhATG8 (pcAb), anti-RhCaspase7 (pcAb), and anti-α-tubulin (mAb) were used as primary antibodies. **(D)** TUNEL staining (red fluorescence) of the tick midgut at different time points following SINV infection. Unfed female *R. haemaphysaloides* infected SINV by microinjection (6 × 10^4^ PFU/per tick), and the midguts were collected by microdissection at different time points after injection (22 tick midguts per time point, 15 tick midguts for RNA extraction, 10 tick midguts for western blot, 2 tick midguts for TUNEL assays).

The cDNAs of tick midguts at different infection time points were subjected to qRT-PCR to evaluate the expression profiles of autophagy- and apoptosis-related genes. To investigate the expression patterns of autophagy related gene (ATG) homologs associated with autophagy, 12 putative *R. haemaphysaloides*-specific ATGs (RhATGs) were identified (Wang et al., 2021). Most RhATGs were upregulated between 1 day and 3 days (early stage) of infection, whereas the transcription levels of apoptosis-related genes did not change significantly in the early stage of infection, but increased significantly with prolonged infection (**Figures 5A, B**). Western blot results showed that autophagy levels were elevated in the tick midgut at the beginning of the infection but decreased later, and apoptosis levels (cleaved-caspase7) increased with prolonged infection (**Figure 5C**). Unfed tick midguts exhibited positive TUNEL staining after infection with SINV-WT at 5 days and 10 days and apoptosis increased over time (**Figure 5D**).

### 3.6 Immune response of tick midguts was activated in the early stage of SINV infection

**Figure 6:**
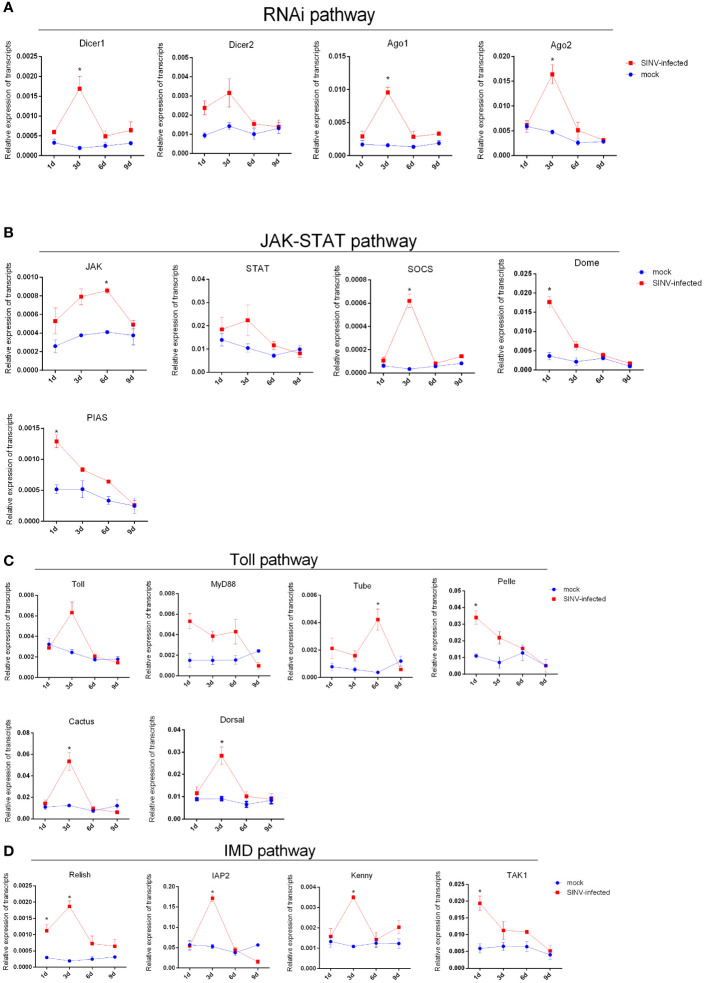
Trends in transcription levels of innate immunity-related genes in ticks during SINV replication in the midguts of unfed female *R. haemaphysaloides*, infected by microinjection. Quantitative qPCR analysis of the expression levels of **(A)** RNAi pathway-related genes. **(B)** Toll-like pathway-related genes. **(C)** JAK-STAT pathway-related gene and **(D)** IMD pathway-related genes after SINV infection. Unfed female *R. haemaphysaloides* infected SINV by microinjection (6 × 10^4^ PFU/per tick), and the tick midguts were collected as mentioned above. Bars represent the mean ± SD of three replicates. Significance of differences as determined by Student’s t-test: *p < 0.05.

After tick infection with a viral load of 6 × 10^4^ PFU/tick by microinjection, the transcription levels of innate immune-related genes (including four innate immune pathways: JAK-STAT, IMD, toll-like, and RNAi interference) were detected by qPCR at different infection time points. All innate immune-related genes were identified using BLAST analysis (Altschul and Lipman, 1990) (http://www.ncbi.nlm.nih.gov/BLAST/). The results showed that the transcription levels of most innate immune-related genes of *R. haemaphysaloides* were significantly elevated during the early stage of infection (infection 1 day–3 days) (**Figures 6A–D**). As the infection time increased, the transcription levels of innate immune-related genes downregulated (**Figures 6A–D**). These results indicate that innate immunity may play a very important role in the early stages of SINV infection in the tick midguts. We found that if the immune storm response fails to clear the virus from *R. haemaphysaloides* in the early stages of SINV infection, SINV will continue to replicate and coexist with *R. haemaphysaloides*, and that viral replication causes disruption of the tick midguts (**Supplementary Figure S3**), which may ultimately lead to the death of the individual tick.

### 3.7 Transcriptional changes in other tick-pathogen interaction molecules reported after SINV infection

**Figure 7:**
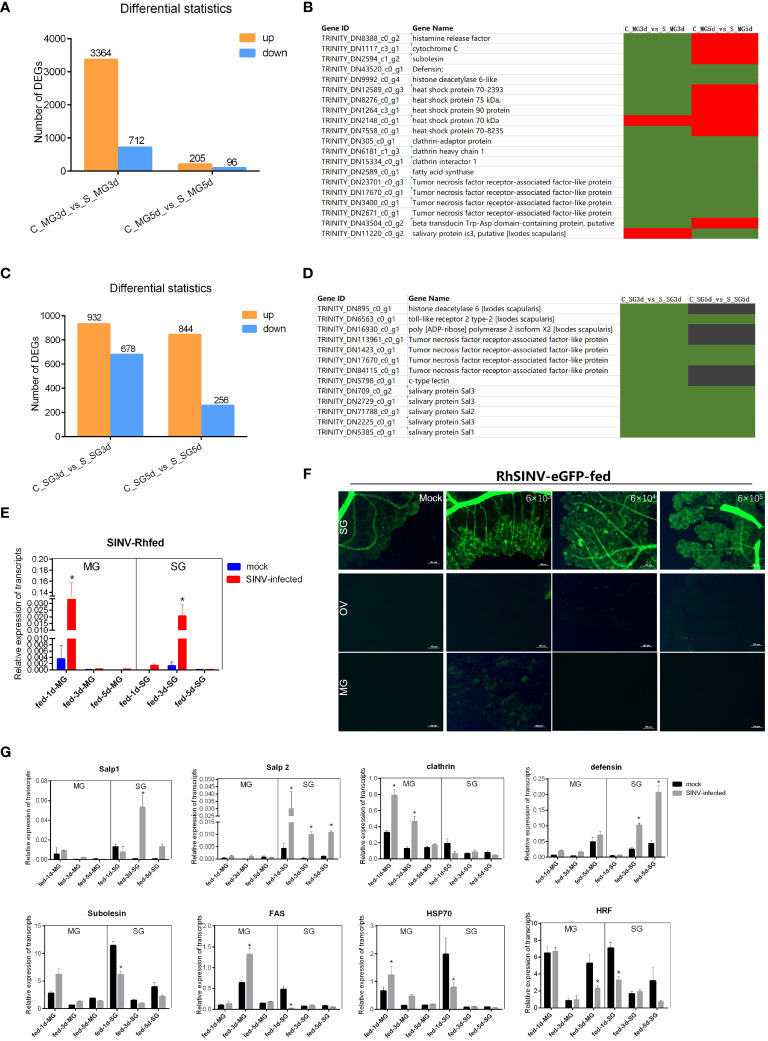
Transcriptome sequencing and differentially expressed gene analysis of the tick midguts and salivary glands at different feeding times of SINV infection by microinjection. **(A)** Differential expressed genes analysis of female *R. haemaphysaloides* midguts transcriptome infected with SINV at different feeding times. **(B, D)** Transcription level analysis of molecules interacting with pathogens in the midguts or salivary glands that have been reported to date. Green indicates upregulated genes with fold change greater than 20 (log2 normalized fold change > 4.32). Black indicates no significant difference. Red indicates downregulated genes with fold change greater than 20 (log2 normalized fold change > 4.32). **(C)** Differentially expressed gene analysis of tick salivary gland transcriptome infected with SINV at different feeding times. **(E)** qRT-PCR detections of SINV in the midgut and salivary glands of fed female *R. haemaphysaloides* at different feeding time points. **(F)** The distribution of SINV in the midguts and salivary glands of fed adult female *R. haemaphysaloides* under a fluorescence microscope; scale bar: 100 μm. **(G)** qRT-PCR detections of tick-pathogen interactions related genes in the midguts and salivary glands of fed female *R. haemaphysaloides* at different feeding time points. Bars represent the mean ± SD of three replicates. Significance of differences as determined by Student’s t-test: *p < 0.05.

To screen for key regulatory molecules involved in SINV transmission, ticks infected by microinjection with SINV were attached to rabbit ears, and the midguts and salivary glands of ticks collected at different feeding time points were analyzed for transcriptome differences. The results showed that the total number of DEGs in the midguts of ticks during early feeding (3 days after attachment) was significantly higher than that on day 5 (**Figure 7A**). We reviewed the articles related to tick-pathogen interactions and screened some of the tick-pathogen interaction molecules with the results of our transcriptome difference analysis. Statistical analysis of the reported transcript levels of molecules interacting with pathogens in tick midguts (Ayllon et al., 2015; Alberdi et al., 2016; de la Fuente et al., 2017; Antunesi et al., 2019) showed that, compared with other pathogens, SINV may have similar mechanisms of infection in tick midguts (**Figure 7B**). The number of DEGs at the two feeding time points in tick salivary glands was basically the same (**Figure 7C**), and changes in the transcription levels of molecules interacting with other pathogens (Ayllon et al., 2015: Alberdi et al., 2016; de la Fuente et al., 2017; Antunesi et al., 2019), were also found in the differential transcriptome analysis of the salivary glands (**Figure 7D**). We examined changes in SINV transcription levels in the midguts and salivary glands at different feeding time points and found that the number of SINV transcripts in the midguts gradually decreased, whereas in the salivary glands, the number of SINV transcripts first increased and then decreased (**Figure 7E**). When adult *R. haemaphysaloides* were infected with SINV-eGFP fed blood, SINV-specific fluorescence disappeared from the fed tick midguts, whereas a large amount of SINV-eGFP-specific fluorescence accumulated in the salivary glands of fed ticks, tentatively demonstrating that SINV-eGFP migrated from the tick midguts to the salivary glands during blood-feeding (**Figure 7F**). We performed qRT-PCR validation of several molecules with clearer functional validation, such as clathrin (Hajdusek et al., 2013; Sonenshine and Macaluso, 2017), HSP70 (Ayllon et al., 2015; Alberdi et al., 2016; Mansfield et al., 2017a), histamine release factor (HRF) (Dai et al., 2010), Salivary glands proteins (Salps) (Liu et al., 2011; Hajdusek et al., 2013; Sonenshine and Macaluso, 2017; Kurokawa et al., 2020; Fogaca et al., 2021) and so on. Based on the above results, some of the DEGs with significant transcription level changes were detected at different transmission time points. The transcription level changes of different genes in the midguts and the trend of the changes in the salivary glands were not the same (**Figure 7G**), which was consistent with the results of the transcriptome difference analysis (**Figures 7B, D**).

## 4 Discussion

In this study, we demonstrated that SINV can be transmitted via ticks and confirmed that SINV replicated in the midguts and salivary glands of ticks both *in vivo* and *in vitro* (**Figures 3A, B** and **Supplementary Figures S1C, D**). Not only these two tick species (*R. haemaphysaloides* and *H. asiaticum*), we also confirmed that SINV replicated in laboratory-reared *Haemaphysalis longicornis* (**Supplementary Figure S4**), and we preliminarily believe that SINV can replicate in most tick species. Therefore, we are interested in knowing whether SINV can replicate in tick cell lines. We found that SINV did not replicate in *I. scapularis* tick embryonic cells (ISE8/CTVM-19). Furthermore, unlike other classical TBVs, such as TBEV (Lindquist and Vapalahti, 2008), POWV (Raney et al., 2022), SFTSV (Zhuang et al., 2018), and CCHFV (Bente et al., 2013), SINV did not replicate in the tick ovaries and the virus was not found in eggs laid by SINV-infected females (**Figure 3F** and **Supplementary Figures S1A, B**). These results indicate that *R. haemaphysaloides* is a competent vector for SINV, but can only transmit the virus in trans-stadial modes. SINV replicated in tick tissues both *in vivo* and *in vitro*, but not in CTVM-19. This indicates that *in vitro* cultures of tick tissues may be closer to the physiological condition inside the ticks, while there are significant differences between tick cell lines and ticks, the reasons for these differences still require further exploration.

To screen the DEGs for SINV-tick interactions, the presence of SINV in ticks was divided into two phases, the replication phase (**Figures 4A–F**) and the phase of SINV transmission (**Figures 7A–D**). We facilitated infection with SINV using microinjection to ensure the stability, accuracy, and representativeness of the screened genes. During the SINV replication stage, we found that the number of DEGs increased significantly with the duration of infection (**Figures 4C, F**). SINV mainly replicated in the midguts of ticks (**Figures 3A, B, E**); therefore, we screened the DEGs in the midguts during the replication period of SINV. Similar to several classic TBVs, such as SFTSV (Xu et al., 2021; Fares and Brennan, 2022), LIV (Johnson, 2017), LGTV (Regmi et al., 2020) and CCHFV (Papa et al., 2017), we found that SINV replication can activate the innate immune responses of ticks (**Figures 4B, E, G** and **Figure 6**). In addition, we also confirmed that SINV infection caused PCD in the tick midguts (**Figures 4B, E, G** and **Figure 5**), which is considered to be a common molecular mechanism for the interactions between ticks and pathogens (Ayllon et al., 2015; de la Fuente et al., 2016b; Mansfield et al., 2017a; Hart and Thangamani, 2021).

For the tick-borne phase of SINV transmission, which involved two main tissues (midguts and salivary glands), we found that the number of DEGs in the midguts of ticks decreased significantly as SINV migrated out of the midguts (**Figure 7A**) and hypothesized that the presence of undifferentiated stem cells in the midguts of ticks is associated with a self-repairing process (Sonenshine, 1991; Parthasarathy and Palli, 2007; Maqbool et al., 2022), whereas the salivary glands of the ticks are highly differentiated cells (Walker et al., 1985; Sonenshine, 1991; Simo et al., 2017) that did not have the ability to self-repair, and that the number of DEGs remained stable despite the decrease in the amount of pathogens (**Figure 7C**). The trends in the expression of genes associated with tick-pathogen interactions were consistent with the results obtained by qRT-PCR. For example, we found that the endocytosis-related gene, clathrin, which mediated migration of *A. phagocytophilum* in ticks (Hajdusek et al., 2013; Sonenshine and Macaluso, 2017), might play an important role in facilitating the passage of SINV across the midgut barrier, but there was no significant change in the transcription levels in the salivary glands. Salps have been reported to play important roles in the tick-borne transmission of several pathogens (such as TBEV, *B. burgdorferi* and *A. phagocytophilum*) (Liu et al., 2011; Hajdusek et al., 2013; Sonenshine and Macaluso, 2017; Kurokawa et al., 2020; Fogaca et al., 2021), and we found significant changes in the transcription levels of several salivary gland proteins during transmission of SINV, but not in the midguts (**Figure 7G**). We also found that tick HRF (critical for the transmission of *B. burgdorferi* in ticks) (Dai et al., 2010) and heat shock protein 70 (HSP70, related to the infection of *A. phagocytophilum*) (Ayllon et al., 2015; Alberdi et al., 2016; Mansfield et al., 2017a) were downregulated during SINV transmission via tick salivary glands, and the mechanism needs to be further investigated.

Beyond that, there were still some limitations of this study. Firstly, it is currently difficult for us to make further comparisons between the differences in physiological responses of ticks induced by SINV and other TBVs due to the lack of relevant studies (Mansfield et al., 2017b; Damian et al., 2020; Maqbool et al., 2022). Secondly, although the PCR positivity rate among adult ticks infected with SINV through feeding reached 100%, it did not correlate with the positivity rates observed in viral plaque assays performed on lysate samples from ticks that tested positive via PCR. Our working hypothesis revolves around the potential insufficiency of the viral load within a single adult tick. This scarcity of virus could impede the formation of virus-specific plaque when the tick’s lysate infects BHK21 cells. This disconnection might explain the disparities between the positivity rates of viral RNA and the presence of virus-specific plaque. The limited formation of virus-specific plaques of BHK-21 cells, infected with blood and brain lysate samples from mice, bitten by SINV-infected adult ticks, may result from a low dose of SINV infection (each mouse can only be infested with 1-2 adult ticks).In contrast, nymphs have the ability to attach to mice in larger quantities (50–100 nymphs per mouse), which represents a high dose of viral infection. As a result, it becomes more probable that tissue lysates of mice bitten by SINV-infected nymphs will generate virus-specific plaques upon infection of BHK21 cells.

To better understand the interactions between ticks and viruses, an increasing number of TBV models have been developed (Maffioli et al., 2014; Grabowski et al., 2018; Zhuang et al., 2018; Salata et al., 2021; Ahmed et al., 2022; Raney et al., 2022). The model based on Langat virus (LGTV) is considered the most widely used, due to its lower biosafety constraints and the close relationship with TBEV and POWV (Kaiser, 2008; Kazimirová et al., 2017; Zhou et al., 2018; Kemenesi and Banyai, 2019; Ahmed et al., 2022; Raney et al., 2022). Although SINV is usually considered a mosquito-borne virus (He et al., 2010; Adouchief et al., 2016), we demonstrated for the first time that SINV can also be transmitted trans-stadially in ticks, just like LGTV (Ahmed et al., 2022). Compared to LGTV, SINV has a more diverse range of virological detection methods and a more comprehensive foundation of virus-related research (Suhrbier et al., 2012; Adouchief et al., 2016; Hameed et al., 2022).

**Figure 8:**
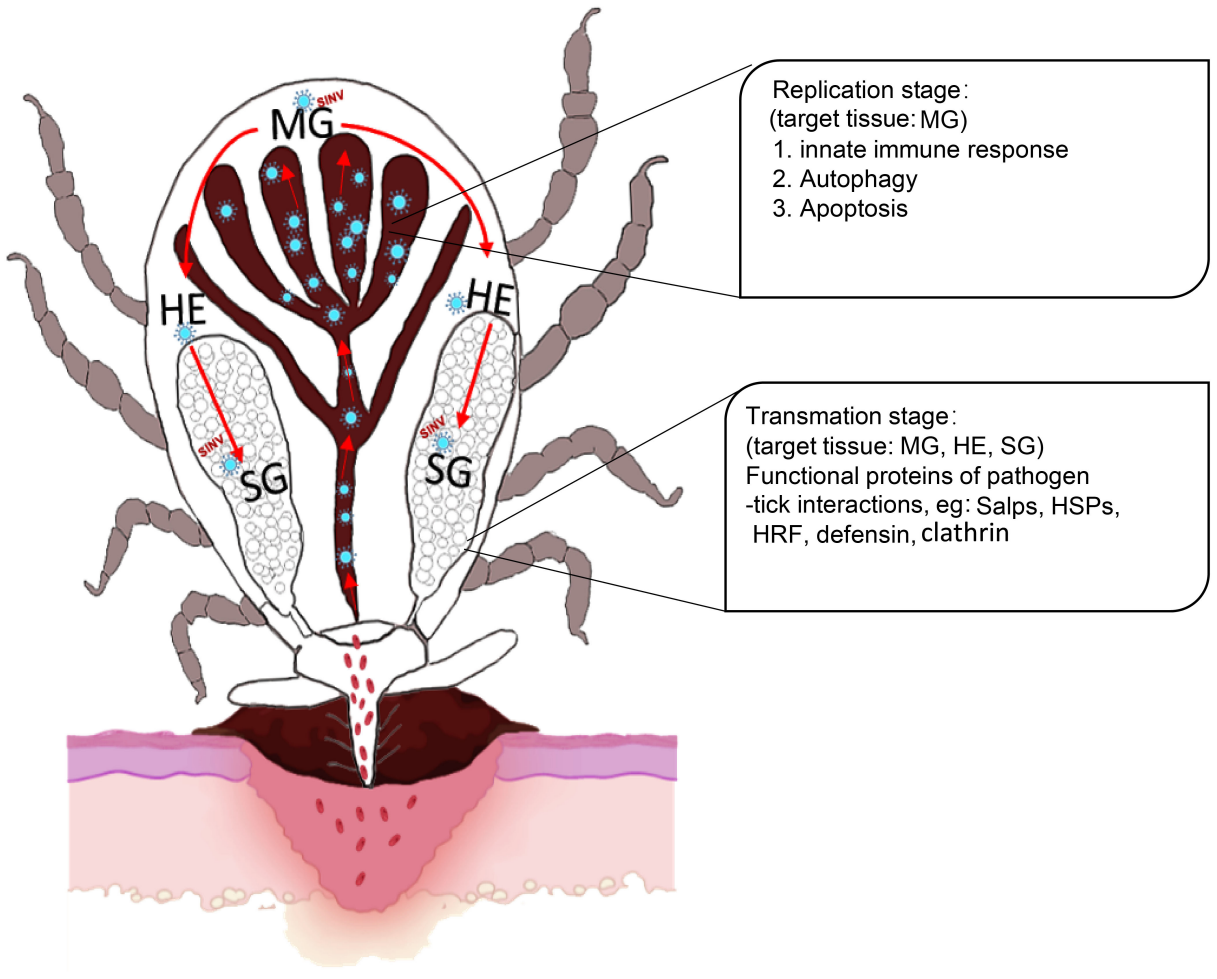
Scheme showing the replication and transmission of SINV in female *R. haemaphysaloides*. The replication stage in the midguts induces autophagy, apoptosis, and innate immune response. Transmission stages (feeding stages) in ticks (Organs involved: midguts, hemolymph, and salivary glands) induce molecules that interact with pathogens in ticks that have been reported to date. MG, midgut; HE, hemolymph; SG, salivary gland.

In summary, we described a new TBV model based on SINV/SINV-eGFP with good biosafety and stability. Firstly, we demonstrated that SINV has a similar transmission pathway to that of other TBPs. Furthermore, combining the results of the transcriptome difference analyses, the presence of SINV in ticks was divided into two phases, the replication phase (involving the midguts, which mainly results in autophagy, apoptosis, and the innate immune response of the midguts), and the transmission phase (involving the midguts, hemolymph, and salivary glands, which mainly exhibit changes in the transcriptional levels of the molecules involved in pathogen interaction in the majority of the ticks that have been reported) (**Figure 8**). Compared to other viruses that can only be transmitted by ticks, we believe that the application of the SINV-based tick-virus interaction model will be more advantageous in understanding the differences between ticks and other vector organisms. Further research is needed to understand the underlying factors that contribute to this difference in replication behavior of SINV in different vectors. This approach allows us to delve deeper into the complex interactions between ticks and viruses. It also provides a new perspective for exploring potential common molecular mechanisms of the interactions between ticks and viruses.

## Data availability statement

The datasets supporting the conclusions of this article are included within the article or uploaded online (https://www.ncbi.nlm.nih.gov/bioproject/1085905; accession numbers: SAMN40349954, SAMN40349955).

## Ethics statement

The animal study was approved by Animal Ethical Committee of Shanghai Veterinary Research Institute. The study was conducted in accordance with the local legislation and institutional requirements.

## Author contributions

YW: Conceptualization, Data curation, Formal analysis, Funding acquisition, Investigation, Methodology, Project administration, Resources, Software, Validation, Visualization, Writing – original draft, Writing – review & editing. ZX: Formal analysis, Investigation, Methodology, Project administration, Software, Validation, Visualization, Writing – original draft. HZ: Data curation, Formal analysis, Investigation, Methodology, Project administration, Writing – original draft. YZ: Data curation, Formal analysis, Methodology, Visualization, Writing – original draft. JC: Methodology, Writing – original draft. YZ: Funding acquisition, Investigation, Methodology, Writing – original draft. ZW: Writing – original draft. JZ: Conceptualization, Data curation, Formal analysis, Investigation, Methodology, Project administration, Resources, Supervision, Writing – review & editing.

## Supplementary material

Supplementary Figure 1 Molecular biology verification of SINV replication in the midguts and salivary glands of unfed female *R. haemaphysaloides in vitro.* **(A)** and **(B)** Distribution of SINV-eGFP in different tissues of unfed female *R. haemaphysaloides* cultured *in vitro* at various infection time points. MG: midgut; SG: salivary gland; OV: ovary; scale bar: 100 μm. **(C)** and **(D)** qRT-PCR to verify the replication of SINV in the eggs and larvae. Bars represent the mean ± SD of three replicates.

Supplementary Figure 2 Analysis of midgut and salivary gland transcriptome differences in ticks infected with SINV at different blood-feeding time points **(A)** Cluster analysis of transcriptome sequencing of the fed female *R. haemaphysaloides.* Midguts and salivary glands at different feeding time points of SINV infection. Histogram of the GO functional annotation of transcriptome DEGs in SINV-infected fed tick midguts **(B)** and salivary glands **(C)**.

Supplementary Figure 3 Fluorescence microscope observation of SINV-eGFP in the tissues of female *R. haemaphysaloides* 360* h* after microinjection. MG: midgut; SG: salivary gland; OV: ovary; scale bar: 100 μm.

Supplementary Figure 4 Fluorescence microscope observation of SINV-eGFP in the tissues of female *H. longicornis* at different time points after microinjection. MG: midgut;.
