# Supplementary material for: Towards modelling tick-virus interactions using the weakly pathogenic Sindbis virus: Evidence that ticks are competent vectors
Source: Front Cell Infect Microbiol. 2024 Mar 19;14:1334351. doi: 10.3389/fcimb.2024.1334351 (PMC10985168; doi:10.3389/fcimb.2024.1334351)
Supplement: Supplementary file 1 [file Table_1.docx]

**S2 Table** Primers for SINV-WT/SINV-eGFP used for qRT-PCR.

| **Primer name** | **Primer sequence** |
| --- | --- |
| SINV nsP1-S | GGTTCCTACCACAGCGACGAT |
| SINV nsP1-A | TGATACTGGTGCTCGGAAAACA |
| probe | FAM-TTGGACATAGGCAGCGCA-MGBNFQ |
| SINV nsP2-S | CTTGTGGTGAAGTCCTACGGTTACG |
| SINV nsP2-A | GTGTGCGGCTGTTGTCTAGTTGT |

**^a^**S, forward primer; A, reverse primer
