# Supplementary material for: Towards modelling tick-virus interactions using the weakly pathogenic Sindbis virus: Evidence that ticks are competent vectors
Source: Front Cell Infect Microbiol. 2024 Mar 19;14:1334351. doi: 10.3389/fcimb.2024.1334351 (PMC10985168; doi:10.3389/fcimb.2024.1334351)
Supplement: Supplementary file 2 [file Table_2.docx]

**S2 Table** Primers for autophagy- and apoptosis-related genes in unfed adult female *R. haemaphysaloides* used for the qRT-PCR analysis.

| **Primer name** | **Primer sequence** |
| --- | --- |
| ELF1A-S | CGTCTACAAGATTGGTGGCATT |
| ELF1A-A | CTCAGTGGTCAGGT TGGCAG |
| RhATG3-S | AGGTGACGAGGATGGCGGCTGGGTT |
| RhATG3-A | ATCCGCATCCAGCAGTCCACTCTCC |
| RhATG4B-S | CCTACCGCAAGAACTTCCCAGCAA |
| RhATG4B-A | TGCCAGTCTTTCCCAAGGTGCCGTC |
| RhATG4D-S | GGCAAGCAGGCGGGTGACTGGTATG |
| RhATG4D-A | TGCCCTTGACGCACGGAATGTATGT |
| RhATG5-S | GAAGCATCGCAGCCAAGTGGTCAGC |
| RhATG5-A | TGTGTGATGATGCTGTGTTTGGCTC |
| RhATG6-S | ACCTGATGTGCCTGGAAGACCCGAC |
| RhATG6-A | GGTAGGGAAGGCAGAAGTTGGAGTC |
| RhATG7-S | TGCCTGAGGAAGTGACCCTTGCCA |
| RhATG7-A | CCCGTTGGCACGACAAGAAAGGAG |
| RhATG8-S | CGCACGCAGTCAACGATAAGCAAGC |
| RhATG8-A | GGTATCTTTGATGGGAACCGTTGCCTG |
| RhATG9-S | CAGTGGGTGCGGAGGTGTCG |
| RhATG9-A | CCGTCCTCACCACTGCCTTCCT |
| RhATG10-S | AGAGCACTGTCTCCGTCATTTGTTC |
| RhATG10-A | TGGAAGTAGGGGACGCCTTGGAT |
| RhATG12-S | CCCCCGAGAAAACCGAAGCCCG |
| RhATG12-A | TTGGCTTCAACCCCAGGCATGCG |
| RhATG14-F | GGGAAGCCATACAACGCACAAGGGA |
| RhATG14-R | GATGTCACGAGCGAGGTCTCCCACG |
| RhATG16-S | GCGGCTGCGGCACACACT |
| RhATG16-A | AATCTCGTTGGCACTGCTCTCTG |
| Rhcaspase1-S | GATGCTGACAGTGGTGTGCCGCC |
| Rhcaspase1-A | CCTGGTTTCGCCCTGAAGTAGAC |
| Rhcaspase-7-S | CTCAGCGAGCGAAGGGGCACGGACA |
| Rhcaspase-7-A | AGCAGACTGGGACAGACATCTCCGT |
| Rhcaspase8-S | CGCCACAGTTTGGGACCACAGGA |
| Rhcaspase8-A | CTTCGCCTTTCACCTGTGCCCCT |
| Rhcaspase9-S | GCTGACAAGCCCACTGGCGAACAAC |
| Rhcaspase9-A | CATTCAGAGCAGAGTCAGCAGTCCG |

**^a^**S, forward primer; A, reverse primer
