# Supplementary material for: Towards modelling tick-virus interactions using the weakly pathogenic Sindbis virus: Evidence that ticks are competent vectors
Source: Front Cell Infect Microbiol. 2024 Mar 19;14:1334351. doi: 10.3389/fcimb.2024.1334351 (PMC10985168; doi:10.3389/fcimb.2024.1334351)
Supplement: Supplementary file 3 [file Table_3.docx]

**S3 Table** Primers for natural immune-related genes of unfed adult female *R. haemaphysaloides* used for qRT-PCR.

| **Primer name** | **Primer sequence** |
| --- | --- |
| RhDicer1-S | GTCGACTTTGTCTTCTGGGTAATA |
| RhDicer1-A | AACTCCGGCTTCCTCATTTC |
| RhDicer2-S | CCTCTCTGGCGGTGAAATAC |
| RhDicer2-A | TTCTGCCTCTTCATCTCAACAA |
| RhAgo1-S | CAGTCTGATGAGGCAGCTTTAC |
| RhAgo1-A | ACGGACCATACCGAAGGATAA |
| RhAgo2-S | CAAGAAGTGATCCAGGCATTAGA |
| RhAgo2-A | CATATTTGGTAGCGGTGCTTTG |
| RhJAK-S | GTGACCTGCAACGAGAAATAGA |
| RhJAK-A | AACTCCATGACCAGCATCAC |
| RhSTAT-S | GGAAGCCGTGAACTCCTTTA |
| RhSTAT-A | TTCTCTGCCTGCTGGATTATG |
| RhSOCS-S | CACAGAGTGGATACTTGGATGG |
| RhSOCS-A | GTGGTAGGTGGCACTGATATG |
| RhDome-S | GCTACACTTTCCACCTGTACTC |
| RhDome-A | GTCTCTGTCTTCGCTGATGTAG |
| RhPIAS-S | CGGGACACTAACTACCAAGATG |
| RhPIAS-A | CAAGACGTCGAAGAAGGGTT |
| RhToll-S | CTCGAAAGCCTCTGGTTTCA |
| RhToll-A | ACCACGGACAATCACGATAA |
| RhMyD88-S | CTTCTCAAGGAACGAGGAAAGA |
| RhMyD88-A | CGTATCTCTCGAGGCACATTAG |
| RhTube-S | TGACTCTGACAGAATGCTCTTC |
| RhTube-A | CGTCGTGCTCCATGTCTTTA |
| RhPelle-S | ACCGACACCTCCTGAATAGA |
| RhPelle-A | AGTGCCTTTGAGCATTGGA |
| RhCactus-S | CAGTGTGCGGGAGATGTATG |
| RhCactus-A | GTCCGTAGTCATTTGGGATGT |
| RhDorsal-S | CACGAAGGACCAGCCATATC |
| RhDorsal-A | TGTCTGGCTTGAGGTGTTG |
| RhRelish-S | GGCTCTCATGGTCCTCTTATTG |
| RhRelish-A | GTGACCAGGCTTGCCTTAAT |
| RhIAP2-S | GTATCTTCTGGACCCTGACCTA |
| RhIAP2-A | ATGGAGGAGCGTTTCCTTTC |
| RhKennγ-S | CAGGAGGTGATCAAAGGTCTAC |
| RhKennγ-A | GATCTTTCTCTCCGGCATTCT |
| RhTAK-S | GACCCAGATGACCAACAACA |
| RhTAK-A | GCACTTCCCACAGGATGATT |

**^a^**S, forward primer; A, reverse primer
