# Supplementary material for: Towards modelling tick-virus interactions using the weakly pathogenic Sindbis virus: Evidence that ticks are competent vectors
Source: Front Cell Infect Microbiol. 2024 Mar 19;14:1334351. doi: 10.3389/fcimb.2024.1334351 (PMC10985168; doi:10.3389/fcimb.2024.1334351)
Supplement: Supplementary file 4 [file Table_4.docx]

**S4 Table** Primers for tick and TBPs interaction-related genes related genes of unfed adult female *R. haemaphysaloides* used for qRT-PCR.

| **Primer name** | **Primer sequence** |
| --- | --- |
| RhHRF-S | TGGAAGGAGGAAGGCAAATC |
| RhHRF-A | CATTGCAGGATTCACCGATAAAG |
| Rhsubolesin-S | TGGAAGGAGGAAGGCAAATC |
| Rhsubolesin-A | CATTGCAGGATTCACCGATAAAG |
| Rhdefensin-S | TGCGTGGACTTTGCATCT |
| Rhdefensin-A | GTGAGCCATCTCGCTTTCT |
| RhHSP-70-S | GACCTCTTCCGTTCCACAAT |
| RhHSP-70-A | CCACCAACCAGCACAATTTC |
| Rhclathrin-S | CTGGCCGACATAAACCAGATAG |
| Rhclathrin -A | CTTTCAGAGGGTCGGTTGTT |
| RhFas-S | CCTCTGGTGTTTGCTCGATAG |
| RhFas-A | GTCGTGCAGAGATGGAATGT |
| RhSal2-S | CACCTTGCTGGAAACATTGG |
| RhSal2-A | TAGCCGAGGCCAGAGTAG |
| RhSal1-S | CGCCTTCGCTGGTTACAT |
| RhSal1-A | CATAGCCGAGGTTGCCATAG |

**^a^**S, forward primer; A, reverse primer
