# Supplementary figures and images for: Towards modelling tick-virus interactions using the weakly pathogenic Sindbis virus: Evidence that ticks are competent vectors
Source: Front Cell Infect Microbiol. 2024 Mar 19;14:1334351. doi: 10.3389/fcimb.2024.1334351 (PMC10985168; doi:10.3389/fcimb.2024.1334351)

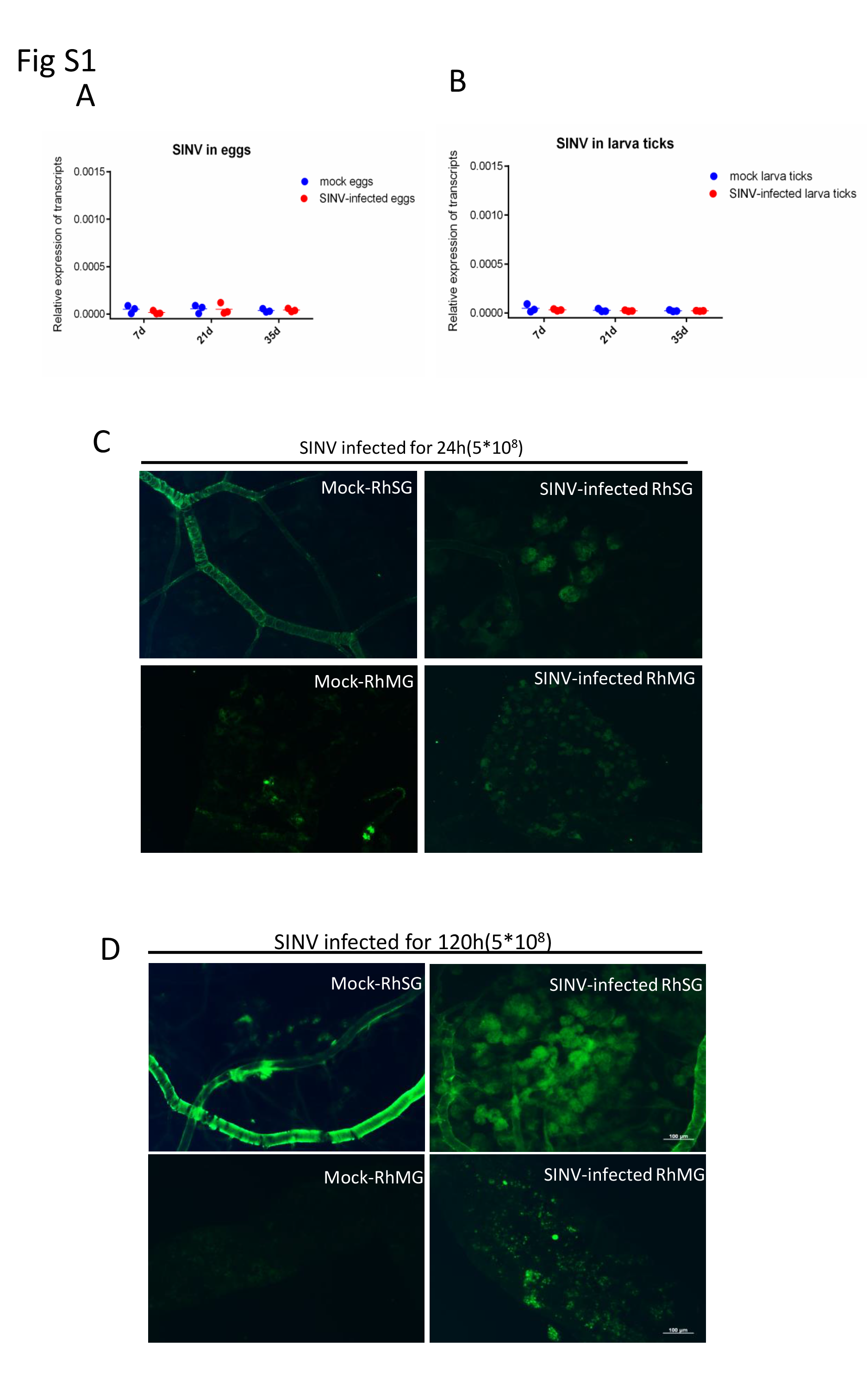

Supplement: Supplementary Figure 1 — Molecular biology verification of SINV replication in the midguts and salivary glands of unfed female R. haemaphysaloides in vitro. (A) and (B) Distribution of SINV-eGFP in different tissues of unfed female R. haemaphysaloides cultured in vitro at various infection time points. MG: midgut; SG: salivary gland; OV: ovary; scale bar: 100 μm. (C) and (D) qRT-PCR to verify the replication of SINV in the eggs and larvae. Bars represent the mean ± SD of three replicates. [file Image_1.tif]

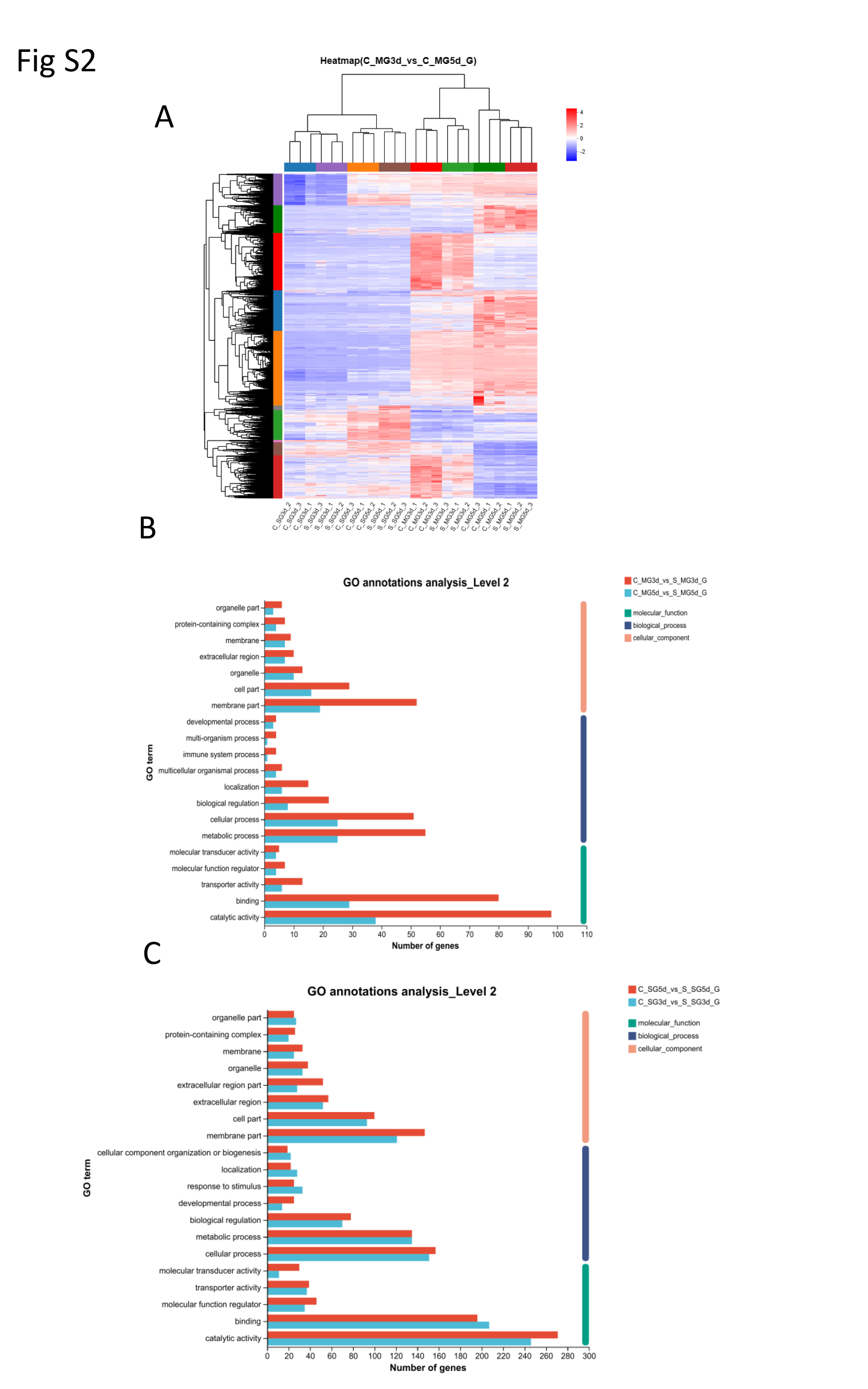

Supplement: Supplementary Figure 2 — Analysis of midgut and salivary gland transcriptome differences in ticks infected with SINV at different blood-feeding time points (A) Cluster analysis of transcriptome sequencing of the fed female R. haemaphysaloides. Midguts and salivary glands at different feeding time points of SINV infection. Histogram of the GO functional annotation of transcriptome DEGs in SINV-infected fed tick midguts (B) and salivary glands (C). [file Image_2.tif]

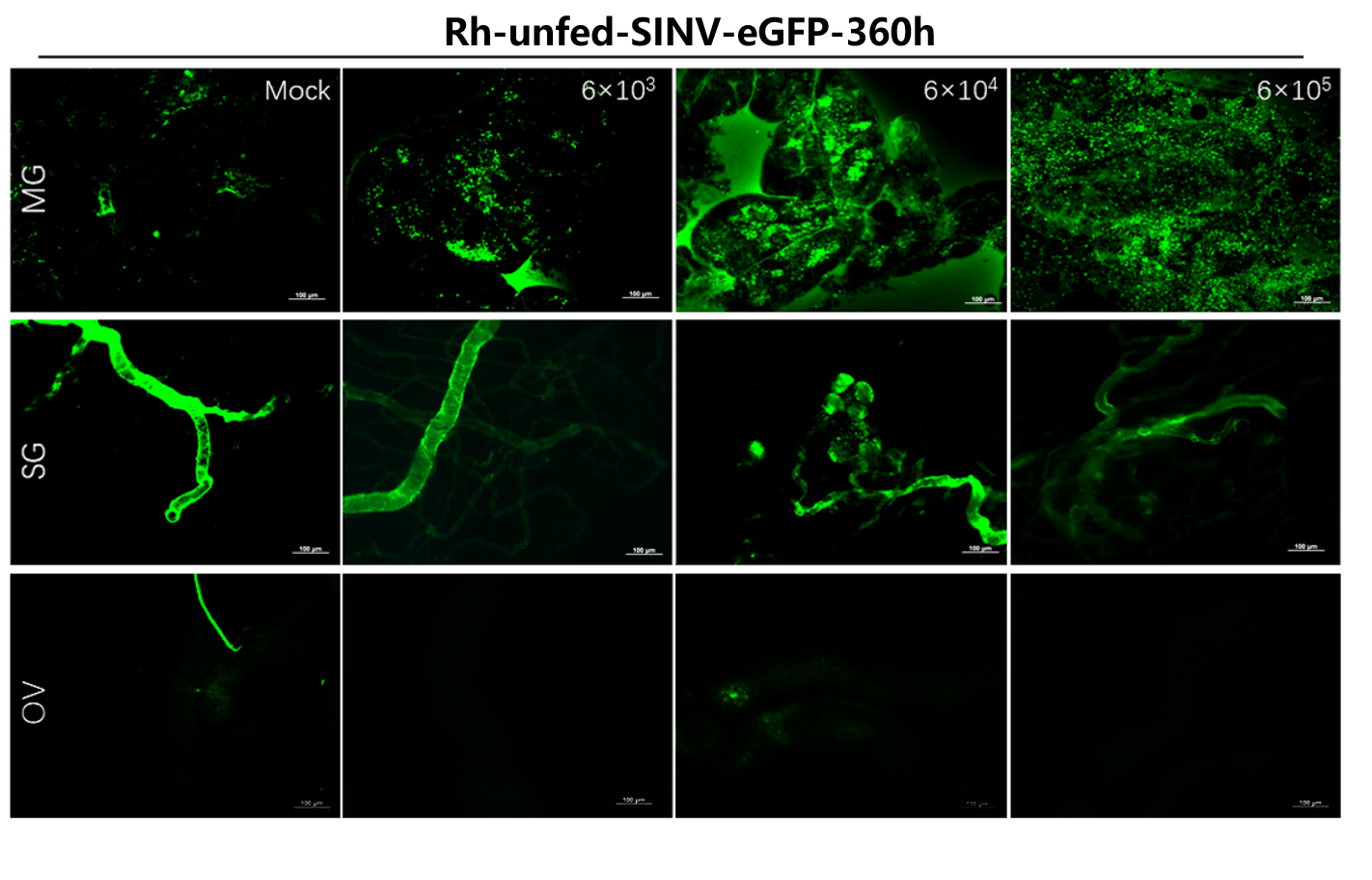

Supplement: Supplementary Figure 3 — Fluorescence microscope observation of SINV-eGFP in the tissues of female R. haemaphysaloides 360 h after microinjection. MG: midgut; SG: salivary gland; OV: ovary; scale bar: 100 μm. [file Image_3.tif]

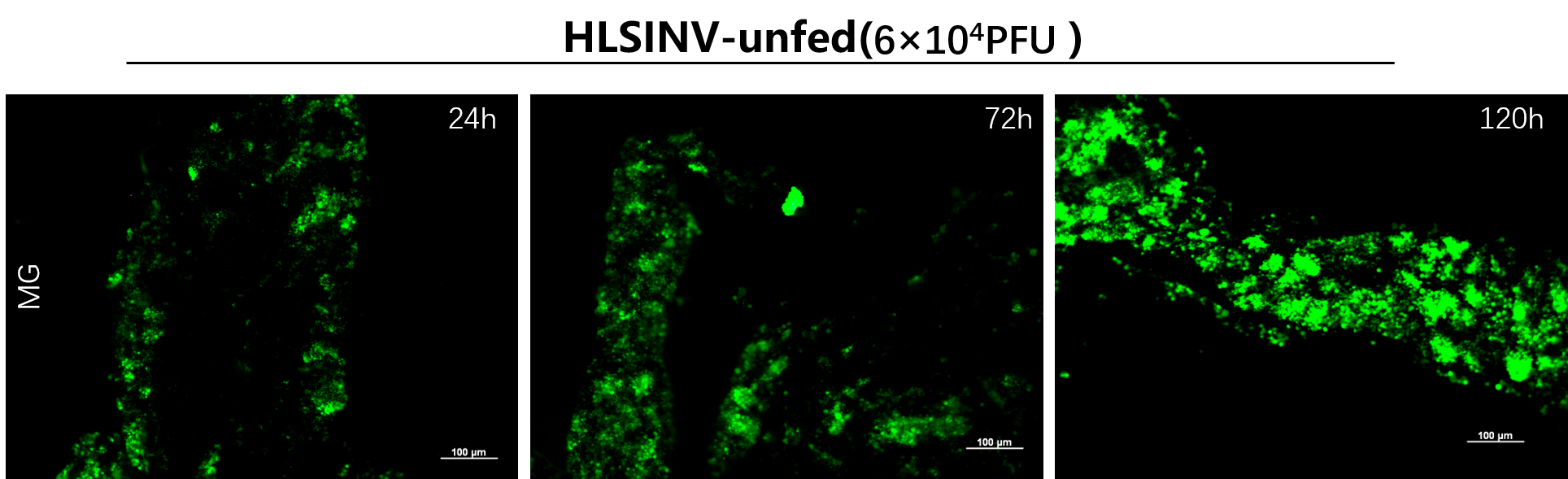

Supplement: Supplementary Figure 4 — Fluorescence microscope observation of SINV-eGFP in the tissues of female H. longicornis at different time points after microinjection. MG: midgut;. [file Image_4.tif]
